# Validating and Calibrating the Nintendo Wii Balance Board to Derive Reliable Center of Pressure Measures

**DOI:** 10.3390/s141018244

**Published:** 2014-09-29

**Authors:** Julia M. Leach, Martina Mancini, Robert J. Peterka, Tamara L. Hayes, Fay B. Horak

**Affiliations:** 1 Department of Biomedical Engineering, Oregon Health & Science University (OHSU), South Waterfront Campus. 3303 SW Bond Avenue, Portland, OR 97239, USA; E-Mail: hayest@ohsu.edu; 2 Department of Neurology, OHSU, Main Campus. 3181 SW Sam Jackson Park Road, Portland, OR 97239, USA; E-Mails: mancinim@ohsu.edu (M.M.); horakf@ohsu.edu (F.B.H.); 3 Department of Biomedical Engineering, OHSU, West Campus. 505 NW 185th Avenue, Beaverton, OR 97006, USA; E-Mail: peterkar@ohsu.edu

**Keywords:** Wii balance board, force plate, validation, calibration, postural sway, balance

## Abstract

The Nintendo Wii balance board (WBB) has generated significant interest in its application as a postural control measurement device in both the clinical and (basic, clinical, and rehabilitation) research domains. Although the WBB has been proposed as an alternative to the “gold standard” laboratory-grade force plate, additional research is necessary before the WBB can be considered a valid and reliable center of pressure (CoP) measurement device. In this study, we used the WBB and a laboratory-grade AMTI force plate (AFP) to simultaneously measure the CoP displacement of a controlled dynamic load, which has not been done before. A one-dimensional inverted pendulum was displaced at several different displacement angles and load heights to simulate a variety of postural sway amplitudes and frequencies (<1 Hz). Twelve WBBs were tested to address the issue of inter-device variability. There was a significant effect of sway amplitude, frequency, and direction on the WBB's CoP measurement error, with an increase in error as both sway amplitude and frequency increased and a significantly greater error in the mediolateral (ML) (compared to the anteroposterior (AP)) sway direction. There was no difference in error across the 12 WBB's, supporting low inter-device variability. A linear calibration procedure was then implemented to correct the WBB's CoP signals and reduce measurement error. There was a significant effect of calibration on the WBB's CoP signal accuracy, with a significant reduction in CoP measurement error (quantified by root-mean-squared error) from 2–6 mm (before calibration) to 0.5–2 mm (after calibration). WBB-based CoP signal calibration also significantly reduced the percent error in derived (time-domain) CoP sway measures, from −10.5% (before calibration) to −0.05% (after calibration) (percent errors averaged across all sway measures and in both sway directions). In this study, we characterized the WBB's CoP measurement error under controlled, dynamic conditions and implemented a linear calibration procedure for WBB CoP signals that is recommended to reduce CoP measurement error and provide more reliable estimates of time-domain CoP measures. Despite our promising results, additional work is necessary to understand how our findings translate to the clinical and rehabilitation research domains. Once the WBB's CoP measurement error is fully characterized in human postural sway (which differs from our simulated postural sway in both amplitude and frequency content), it may be used to measure CoP displacement in situations where lower accuracy and precision is acceptable.

## 1. Introduction

The Wii balance board (WBB) (Nintendo, Kyoto, Japan) has generated significant interest beyond the public domain, particularly in its application as a postural control measurement device in both the clinical and (basic, clinical, and rehabilitation) research domains. Postural control is an essential daily life function that can be measured by characterizing postural sway (the postural shifts in both the anteroposterior (AP) and mediolaterial (ML) directions during quiet stance). Postural control is a complex motor function derived from the integration of several neural components including sensory and movement strategies, orientation in space, biomechanical constraints, and cognitive processing [1]. When one or more of these components are compromised, postural instabilities arise.

Postural instability is one of the most common causes of dependence, reduced quality of life, and falls, a leading cause of injury and subsequent death for older adults [2–4]. Because both direct and indirect costs of postural instability are significant, rising, and increasingly unsustainable for our healthcare system, there is a pressing need to monitor, manage, and help improve postural stability in our aging population [3,5]. This need may be addressed via preventative and therapeutic health care practices that promote frequent assessments of postural sway. Frequent, longitudinal postural sway monitoring may enable early detection of motor decline, cognitive decline, and/or elevated fall risk and in turn may yield opportunities for intervention, treatment, compensation, coping, sustained independence, and prevention of irreversible damage. However, first, a means to frequently and longitudinally measure postural sway must be identified, validated, and implemented.

Posturography is the traditional instrumental technique used to objectively quantify postural sway. This technique uses one or two laboratory-grade force plates to measure two-dimensional center of pressure (CoP) displacement. Prior research has shown force plate-based CoP measures to be sensitive to mild postural instability in older adults with mild neurodegenerative diseases and/or a high fall risk [6–14]. However, because force plates are expensive, not easily portable, and require proper installation, they are not feasible for quantifying postural sway in the small clinic or home on a frequent basis. Although, frequently quantifying postural sway in the laboratory is neither reasonable nor economical. The WBB has been recently proposed as an affordable, portable, and easily accessible alternative to the force plate [15–20], however additional research is necessary before the WBB can be considered a valid and reliable CoP measurement device.

Both the WBB and laboratory-grade force plate measure force distribution and the resultant CoP displacement. However, there are significant differences between devices, pertaining to both material composition and technical capacity, which result in functional limitations of the WBB. Force plates are composed of metal while the WBB is composed of plastic. Due to the WBB's material properties, it is susceptible to elastic deformation when a significant load is applied to the WBB's usable surface. If the usable surface deforms during data acquisition, the WBB's ability to acquire accurate CoP measurements may be hindered. Also, both devices rely on four force sensors located near each of the four corners of the plate or board to measure force distribution. Force plates measure tri-axial forces and moments while the WBB only measures uni-axial (vertical) forces. Because the WBB is unable to measure moments and horizontal forces, its ability to acquire accurate CoP measurements may be hindered when the input signal has significant horizontal and shear components. The WBB's accuracy is further restricted by several mechanical and electronic limitations, characterized in a 2011 publication on the differences between the WBB and a force plate. Pagnacco *et al.* [21] clearly substantiated the WBB's low resolution (0.5 mm), low and inconsistent sample rate (time jitter), low signal to noise ratio, and occasional glitches in the WBB data (discussed further in Section 5). According to the authors, a significant amount of noise in the WBB data can be attributed to the unshielded cables, under-designed electronics (incapable of noise minimization), and unsynchronized sampling across the four force sensors. These limitations, along with the uncertain validity and reliability of WBB-based CoP measures derived from a dynamic input signal, currently restrict our utilization of the WBB for clinical or research purposes.

Many studies have used the WBB to quantify postural sway in varying populations (e.g., healthy young, healthy old, and impaired old) and under varying sway conditions (e.g., eyes open *vs.* eyes closed, single- *vs.* double-leg stance, *etc.*) [15–19]. In all but two prior studies [16,21], the WBB and force plate were used to measure CoP displacement during separate trials. Although WBB- and force plate-based CoP measures were found to be highly correlated, CoP measurement error could not be determined since CoP displacement was not measured simultaneously by the WBB and force plate. In 2011, Pagnacco *et al.* [21] were the first to simultaneously measure CoP displacement with the WBB and force plate, eliminating within-subject variability and increasing the validity of their between-device comparison. Unlike in previous work [15], Pagnacco and colleagues chose to not calibrate the WBB data using a custom calibration method and used the manufacturer's internally-stored values instead. The authors relied on the WBB's internal calibration values *vs.* those determined empirically because a custom calibration method is expensive, time intensive, and neither affordable nor feasible for most users. Also, according to Pagnacco *et al.*, custom calibration detailed in Clark *et al.* [15] has minimal effect on the noise inherent in the WBB data. For data acquisition, the authors quantified the WBB's CoP measurement error for two “subjects”—A 50 kg dead weight and a 48 kg, 1600 mm tall human—During 60 s of quiet stance. In doing so, Pagnacco and colleagues characterized the WBB's mechanical and electronic limitations as a CoP measurement device (discussed above). Despite the aforementioned limitations and Pagnacco's strong recommendation to not use the WBB for anything other than its intended use (*i.e.*, as a toy) [21,22], the WBB continued to generate significant interest in both the clinical and research domains.

In 2013, Huurnink *et al.* [16] were the second to measure CoP displacement with the WBB and force plate simultaneously. The authors investigated postural sway in 14 healthy, young adults under three different sway conditions (single-leg stance with eyes open, with eyes closed, and after a short sideways hop). Although Huurnink and colleagues determined the WBB as “sufficiently accurate” when measuring CoP displacement, they only investigated a narrow CoP displacement range (*i.e*., which was restricted to the area of the standing footprint during their single-leg stance conditions) and quantified CoP using only two, two-dimensional time-domain measures (mean sway amplitude and velocity). Because Huurnink *et al.* did not assess one-dimensional (AP *vs.* ML) CoP measures, they were unable to quantify the WBB's dimension-specific performance error (e.g., the WBB may be more accurate in measuring sway in the AP direction compared to that in the ML direction). Additionally, the authors did not assess the WBB's ability to measure frequency content, nor did they assess the inter-device variability across multiple WBBs [16].

Information typically available for laboratory-grade force plates, such as measurement uncertainty and reliability across varying sway conditions and measurement variability across multiple devices, was unavailable for the WBB until a recent 2014 publication by Bartlett *et al.* [20]. Bartlett and colleagues conducted a standard measurement uncertainty analysis to quantify the repeatability and accuracy of WBB CoP measurements. They also assessed the effect of wear (lightly used *vs.* heavily used WBBs) on CoP measurement performance. Two different static loads (14.3 kg and 45.8 kg) were systematically applied to five specified locations on the WBB's usable surface (center and four corner positions located approximately halfway from the WBB's center to the corner edges). Nine WBBs (three lightly used, six heavily used) were tested. The authors found the total uncertainty of CoP measurement to be within ±4.1 mm across the nine WBBs, which is much higher than that recommended for posturography (0.1 mm). They found repeatability within a single WBB to be better (1.5 mm), suggesting that the WBB be applied as a relative (*vs.* absolute) CoP measurement device (*i.e.*, comparing measurements within, as opposed to across, WBBs). Consistent with previous findings [23], Bartlett *et al.* found the WBB to behave linearly, with a statistically significant increase in error from the center to the corner locations and from the light to heavy static loads. There was no significant effect of wear on mean CoP measurement error. Additionally, the authors found the WBB's internal calibration values to be comparable to those determined empirically. According to Bartlett *et al.*, although the WBB lacks the accuracy recommended for posturography and should not be used as a replacement for the “gold standard” laboratory grade force-plate, it may be used to estimate force and CoP measurements when lower accuracy and precision is acceptable [20]. In static analyses, the WBB may be sensitive to postural sway differences greater than 10 mm, which could differentiate between healthy and impaired populations [20,24].

Although Bartlett *et al.* clearly specified the WBB's limitations when measuring CoP under controlled static conditions, the WBB's CoP measurement error under controlled dynamic conditions remains unknown. Characterizing the WBB's performance under controlled dynamic conditions is imperative since most, if not all, potential WBB applications call for measuring biomedical signals which are dynamic by nature. As mentioned above, the WBB has been used to measure CoP in many different human populations and under a variety of postural sway conditions in an effort to test the WBB across varying sway profiles. Nonetheless, human sway remains an uncontrolled input signal, rendering the experimenter unable to systematically test the WBB's CoP measurement error with respect to specific postural sway features (e.g., sway amplitude, frequency, velocity, *etc.*). Quantifying the WBB's CoP measurement error with controlled, dynamic input/output signals is fundamental in our effort to fully characterize the WBB's limitations as a CoP measurement device.

In this study, we used the WBB and a laboratory-grade force plate (AFP) (AMTI OR6-6, Watertown, MA, USA) to simultaneously measure one-dimensional CoP displacement of controlled, dynamic input/output signals. An inverted pendulum mechanical system was employed as our dynamic load so we could systematically modulate CoP displacement (*via* adjustments made to the inverted pendulum's displacement angle and load height). The WBB's CoP measurement error was quantified and analyzed with respect to sway amplitude, frequency, and direction (AP *vs.* ML). Twelve WBBs were tested to address the issue of inter-device variability. Our two research aims were: Aim I, to validate the WBB against the “gold standard” AFP by quantifying the WBB's CoP measurement error under controlled dynamic conditions; and, Aim II, to determine the WBB's inter-device variability across 12 different WBBs.

## 2. Experimental Methods

**Figure 1.**
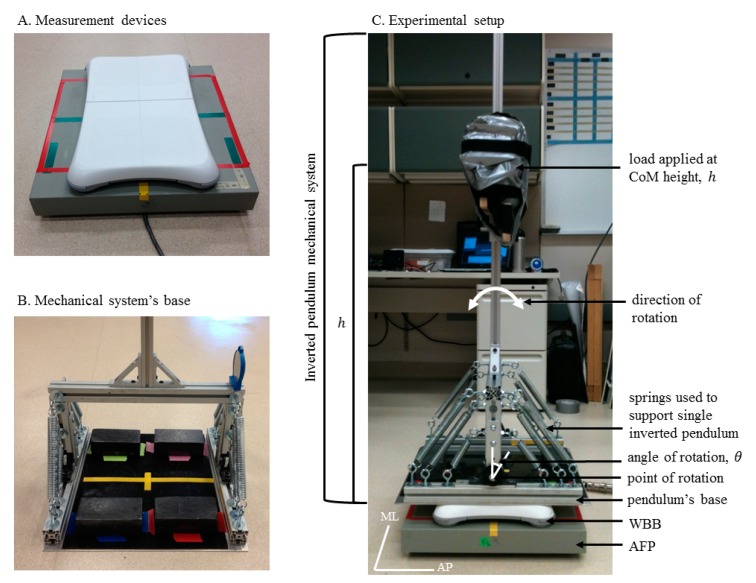
Experimental setup to measure simulated one-dimensional postural sway. (**A**) The Nintendo Wii balance board (WBB) mounted and centered on the AMTI force plate (AFP); (**B**) Four (6.8 kg) lead blocks positioned symmetrically on the mechanical system's base to stabilize the inverted pendulum during oscillation; (**C**) The experimental setup: the mechanical system was mounted and centered on the WBB, which was mounted and centered on the AFP. The mechanical system consisted of a single inverted pendulum supported by springs (15.1 kg), a (16.0 kg) load applied at the CoM height, *h*, and four lead blocks positioned on the base to stabilize the inverted pendulum during oscillation. The inverted pendulum was displaced at a specified angle, *θ_i_*, and then released to oscillate in the AP direction. The mechanical system was rotated 90° to acquire one-dimensional sway in the ML direction.

Our experiment was conducted under controlled laboratory conditions using an inverted pendulum mechanical system (described in Section 2.1 and illustrated in Figure 1) to simulate one-dimensional postural sway. We carried out one laboratory experiment to address our two research aims. For Aim I, we tested a variety of sway amplitudes and frequencies in both sway directions to validate the WBB against the AFP. For Aim II, we repeated our Aim I testing protocol (detailed in Section 2.2) with 12 different WBBs to determine the WBB's inter-device variability. All data were collected at the Oregon Health & Science University using resources and materials from the Balance Disorder's Laboratory and the Human Spatial Orientation Laboratory.

### 2.1. Description of Mechanical System

A single inverted pendulum mechanical system (Figure 1C) was constructed to simulate one-dimensional postural sway. Springs (Figure 1C) were employed to counteract gravitational forces and stabilize the pendulum at equilibrium (perpendicular to the ground). The inverted pendulum weighed 15.1 kg, with most of its weight concentrated at the base. The pendulum was loaded to the maximum tolerable weight (16.0 kg) at the approximate height of a human body's center of mass (CoM) [25,26]. Four lead blocks, each weighing ∼6.8 kg, were then positioned symmetrically on the pendulum's base (Figure 1B) to stabilize the loaded pendulum as it oscillated. Therefore, the total mass of the mechanical system was 15.1 + 16.0 + 4 × 6.8 = 58.3 kg. To simulate one-dimensional postural sway, the inverted pendulum was displaced at a specified angle and then released. The pendulum oscillated, following a dampened oscillation pattern due to internal friction and air resistance. To test a variety of sway amplitudes and frequencies (for Aim I), we systematically adjusted both the displacement angle and load height prior to each trial.

### 2.2. Procedures

Aim I: To validate the WBB against the AFP
  The mechanical system was mounted and centered on the WBB (Figure 1C), which was mounted and centered on the AFP (Figure 1A). Our testing protocol consisted of nine 30-second trials to test a variety of sway amplitudes and frequencies: three initial displacement angles (*θ_i_* = 2°, 4° and 6°) at three different load heights *h* = 900, 1000 and 1100 mm, corresponding to three different oscillation frequencies (*ω*) = 0.6, 0.5, and 0.4 Hz, respectively). Because the pendulum oscillated in one-dimension, the testing protocol was repeated twice to acquire sway data in both the AP and ML directions, for a total of 18 30-s trials for each WBB. (NOTE: The mechanical system was rotated 90° to acquire sway data in the ML direction (Figure 1C)).
Aim II: To determine the WBB's inter-device variability
  The Aim I testing protocol detailed above was repeated 12 times with 12 different WBBs. Two WBBs had been lightly used and the remaining 10 were new.

**Figure 2.**
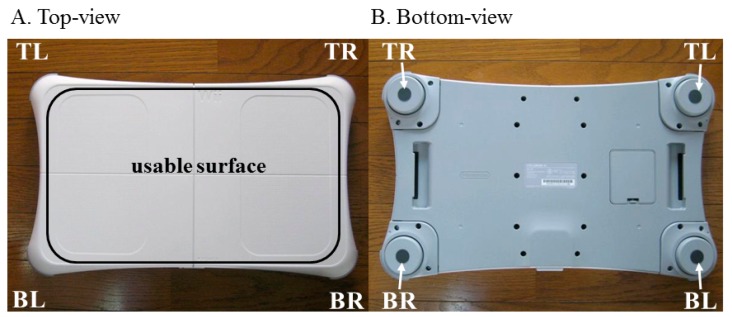
The Nintendo WBB. (**A**) Top-view of the WBB shows the usable surface; (**B**) Bottom-view of the WBB shows the four foot-pegs, located under each of the four corners of the WBB: top right (TR), top left (TL), bottom left (BL), and bottom right (BR). The four force sensors are housed in the four foot-pegs.

CoP displacement was measured by both an AFP and a WBB. The WBB functions with four force sensors housed in the foot-pegs located under each of the four corners of the WBB (Figure 2B). The force sensors act as uni-axial force transducers, each consisting of a metal beam and strain gauge, and measure vertical forces [20]. The WBB was interfaced with a laptop computer (operating on Microsoft Windows Vista) using custom-written software (C++) and a Bluetooth connection. The initial (vertical) offset was recorded by each of the four force transducers when the WBB was first connected, before the mechanical system was positioned atop the WBB's usable surface (Figure 2A). During data acquisition, both raw sensor values and internal calibration values (issued at three different calibration points) were reported for each of the four force transducers. The raw sensors values were converted into calibrated mass measurements (in kg) using the internal calibration values and the initial (vertical) offset and were then converted into force units (N). (The use of the manufacturer's internally-stored calibration values to calibrate WBB data is justified in by Pagnacco *et al.* [21], detailed in Section 1). The calibrated sensor values were then stored as our WBB data. The AFP was calibrated in accordance with the manufacturer's recommendations. The initial (tri-axial) offset was recorded by the AFP prior to data acquisition, when the WBB was mounted and centered on the AFP, but before the mechanical system was positioned atop both measurement devices. The weight of the WBB was subtracted from the vertical force channel of the AFP's initial offset. This adjusted offset, excluding the vertical force applied by the weight of the WBB, was then used to calibrate the AFP measurements. Calibrated tri-axial forces and moments were stored as our AFP data.

### 2.3. Data Acquisition

To determine an appropriate sampling rate, the spectral characteristics of our simulated postural sway were first examined. During pilot testing, the inverted pendulum's maximum oscillation frequency (induced by the shortest load height) was found to be 0.6 Hz. All frequency content within the power spectrum lay below 1.0 Hz for all tested displacement angles and load heights.

The WBB sampled at approximately 50 Hz when interfaced with our laptop computer. Because the WBB samples at an inconsistent rate, a data averaging method was employed to create time series with samples at equal time intervals (*t_DA_*). During data acquisition, our custom-written software averaged across (approximately 3–6) samples every 93.75 ms (*t_DA_* = 0.09375 s; data averaging frequency, *f_DA_* = 1/*t_DA_* = ∼10.7 Hz). Although a rate of ∼10.7 Hz is low compared to what is clinically recommended for posturography [27], it was high enough to capture the spectral characteristics of our simulated postural sway since all frequency content lay below 1.0 Hz.

The AFP sampled at 100 Hz, and a 10.5 Hz low-pass filter was applied during data acquisition.

### 2.4. Data Analysis

All data were analyzed in Matlab R2014a (The MathWorks, Natick, MA, USA).

#### 2.4.1. CoP Signals

To account for the inherent (yet small) positioning errors during the experimental setup (described in Section 2.2: The mechanical system was mounted and centered atop the WBB, which was mounted and centered atop the AFP), a Principal Component Analysis [28] was used to transform the (*x*- and *y*-) axes of both the WBB and AFP datasets. CoP displacement (in both the AP (*y*-axis) and ML (*x*-axis) directions) was then calculated from the transformed axes of both WBB and AFP data.

##### WBB-Based CoP Signals

**Figure 3.**
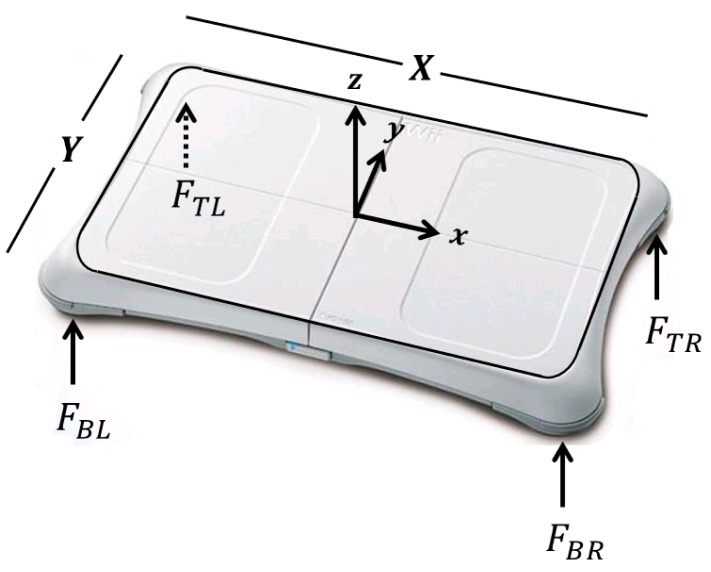
A diagram of the WBB. Accurate *X* and *Y* dimensions are essential for accurate CoP calculations: *X* = 433 mm, *Y* = 238 mm.

The WBB measures vertical (*z*-axis) ground reaction forces but is unable to measure horizontal (*x*- or *y*-axis) forces and (*x*-, *y*-, and *z*-axis) moments (Figure 3). Specifically, the CoP calculations used for the WBB data do not take horizontal and shear components into account. The WBB's calibrated sensor values (discussed in Section 2.2) were expressed in force units (N). The vertical forces (*F_TR_, F_BR_, F_TL_, F_BL_)* measured by each of the four force transducers were then used to calculate CoP for the WBB (*CoP_WBB_*):

(1) $$\begin{array}{cc} {\text{CoP}}_{{\text{WBB}}_{x}}=\frac{X}{2}\frac{\left(F_{TR}+F_{BR})-(F_{TL}+F_{BL}\right)}{F_{TR}+F_{BR}+F_{TL}+F_{BL}}; & {\text{CoP}}_{{\text{WBB}}_{y}}=\frac{Y}{2}\frac{\left(F_{TR}+F_{TL})-(F_{BR}+F_{BL}\right)}{F_{TR}+F_{BR}+F_{TL}+F_{BL}} \end{array}$$

where *X* and *Y* represent the distance (in mm) between each force transducer assuming that each transducer is positioned in the center of each foot-peg, and *CoP_WBBx_* and *CoP_WBBy_* represent the CoP displacement (in mm) calculated in the ML and AP directions, respectively [29].

##### AFP-Based CoP Signals

**Figure 4.**
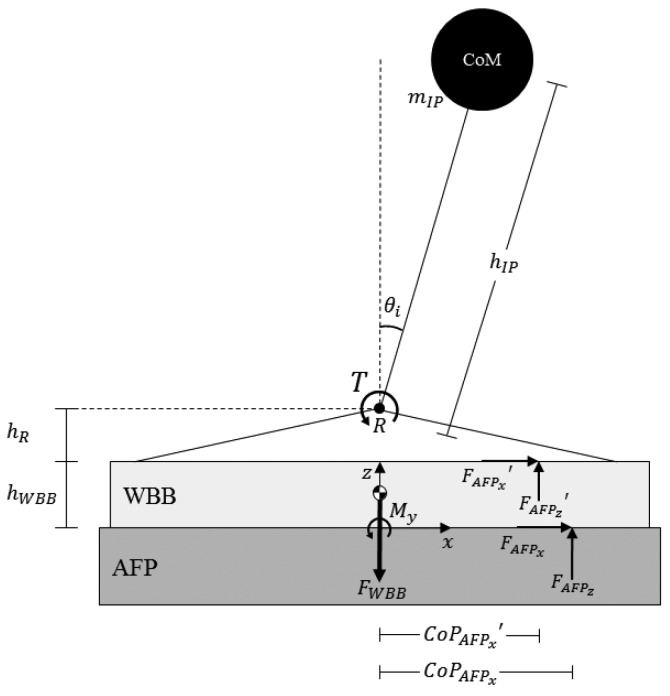
A simplified diagram of the experimental setup for *x*-direction CoP displacement. (NOTE: *F_AFPx_′* = *F_AFPx_* since these values reflect the acceleration of the CoM, which is the same for both the WBB and AFP).

The AFP measures tri-axial (*x*-, *y*-, and *z*-axis) forces (*F*) and moments (*M*) (*F_x_, F_y_, F_z_, M_x_, M_y_*, and *M_z_*), providing the “gold standard” measurement of CoP. For our experimental setup, a given motion of the inverted pendulum produced a different CoP displacement at the surface of the WBB compared to the surface of the AFP due to: 1. the additional height of the WBB (*h_WBB_*), and 2. the additional (static) force applied to the surface of the AFP from the weight of the WBB (*F_WBB_*) (Figure 4). In order to compare CoP measured by the AFP to that measured by the WBB (*CoP_WBB_*), a CoP prediction (*CoP_AFP_′* and in Figure 4) of the CoP at the surface of the WBB was derived from AFP data and known parameters of the experimental setup.

First, *x*-direction CoP displacement was calculated (*CoP_AFPx_*) in accordance with AMTI Biomechanics Platform Instructions Manual. Then, the predicted CoP at the WBB surface (*CoP_AFPx_′*) was calculated using the following procedure:

1. With known *CoP_AFPx_*, moments were summed about the point of rotation, *R* to calculate *T*:
  (2) $$T={\text{CoP}}_{{\text{AFP}}_{x}}F_{{\text{AFP}}_{z}}+(h_{R}+h_{\text{WBB}})F_{{\text{AFP}}_{x}}$$
2. With known *T*, the AFP's prediction of the WBB's CoP measurement was calculated:
  (3) $${{\text{CoP}}_{{\text{AFP}}_{x}}}^{\prime }=[T-h_{R}{F_{{\text{AFP}}_{x}}}^{\prime }]/{F_{{\text{AFP}}_{z}}}^{\prime }$$
  where *F_AFPx_′* = *F_AFPx_, F_AFPz_′* = *F_AFPz_* − *F_WBB_, F_WBB_* = *m_WBB_g, m_WBB_* = the mass of the WBB, and *g* = acceleration due to gravity.

A similar calculation was made for CoP displacement in the y-direction. The AFP-derived signals *CoP_AFPx_′* and *CoP_AFPy_′* were then compared to the WBB-based CoP (*CoP_WBBx_* and *CoP_WBBy_*) obtained using Equation (1).

##### Influence of Displacement Angle and Load Height on CoP Displacement

As expressed in Equation (3) and illustrated in Figure 4, the AFP's prediction of the WBB's CoP displacement = *CoP_AFPx_′* = [*T* − *h_R_F_AFPx_′*]/*F_AFPz_′*. The distance from *R* to the surface of the WBB (*h_R_*) is a constant and the vertical force (*F_AFPx_′*) is unaffected by changes in both displacement angle (*θ*) and load height (*h*). So, CoP displacement is dependent on both *T* and the horizontal force (*F_AFPx_′*), which are both functions of *θ_i_* and *h*:

(4) $$T(t)=(k-m_{IP}gh)\theta (t)$$

where *k* = the spring constant of springs supporting the inverted pendulum, *m_IP_* = the mass of the inverted pendulum mechanical system, *h* = the height of *m_IP_* above the rotation axis, and *t* = time. Ignoring any damping, the angular motion of the inverted pendulum is given by:

(5) $$\theta (t)=\theta_{i}\sin(\omega t)$$

where *ω* is the oscillation frequency of the inverted pendulum, and *θ_i_* = the initial angular displacement of the pendulum. From torsion pendulum mechanics, the oscillation frequency is given by:

(6) $$\omega =\sqrt{(k-m_{IP}gh)/m_{IP}h^{2}}$$

thus as *h* increases *ω* decreases.

The horizontal shear is proportional to the horizontal acceleration of the pendulum mass:

(7) $${F_{{\text{AFP}}_{x}}}^{\prime }=-m_{IP}ẍ\approx -m_{IP}h\ddot{\theta }(t)=m_{IP}h\theta_{i}\omega^{2}\text{sin}(\omega t)$$

so,

(8) $${{\text{CoP}}_{{\text{AFP}}_{x}}}^{\prime }(t)=[(k-m_{IP}gh-h_{R}m_{IP}h\omega^{2})\theta_{i}\sin(\omega t)]/{F_{{\text{AFP}}_{z}}}^{\prime }$$

therefore, the main effect is that larger *θ_i_* produces larger *CoP_AFPx_′* but a secondary effect is that a shorter load height *h* also produces a larger *CoP_AFPx_′*.

##### CoP Signal Processing

All CoP signals were low-pass filtered with a fourth-order, zero-phase Butterworth filter with a cutoff frequency of 5 Hz [30]. Because the WBB sampled at a different rate than the AFP, the *CoP_WBB_* signals were resampled at 100 Hz to match the *CoP_AFP_′* sampling rate. Additionally, because the WBB and AFP were not time-aligned during data acquisition, offline signal synchronization was necessary. To synchronize offline, the *CoP_WBB_* and *CoP_AFP_′* signals were zero-meaned, cross-correlated using a Hanning window, and time-aligned.

#### 2.4.2. CoP measures

Time- and frequency-domain CoP measures (Table 1) were derived from the last 25 s of both the *CoP_WBB_* and *CoP_AFP_′* signals. The calculations of the time-domain measures are detailed in Prieto *et al.* [30]. The single frequency-domain measure, peak frequency, was determined by finding the frequency index of the power spectrum at which the maximum power lies. The power spectrum was estimated using Welch's method [28].

#### 2.4.3. Quantifying the WBB's Performance by Determining CoP Measurement Error

The CoP measurement error was differentiated into two parts: CoP signal error and CoP measure error. The CoP signal error (defined below) pertains to the difference between the *CoP_WBB_* and *CoP_AFP_′* signals. The CoP measure error (defined below) pertains to the difference between the WBB- and AFP-based CoP measures defined in Table 1.

The WBB's performance was first quantified by comparing the CoP signals (*CoP_WBB_vs. CoP_AFP_′)*. The CoP signal error was defined as the difference (in mm) between the WBB CoP measurement and the AFP CoP measurement (*CoP_WBB_* − *CoP_AFP_′)*. This error value was calculated for each data point in every trial (across all sway amplitudes and in both directions) for each WBB. Agreement between measurement devices (AFP *vs.* WBB) was visually represented by plotting the CoP signal error against the “gold standard” AFP CoP measurement (*CoP_AFP_′*). Simple linear regression was used to fit a straight trend line to the CoP signal error plotted against the *CoP_AFP_′* signals: *CoP_WBB_* − *CoP_AFP_′* = *β* × *CoP_AFP_′* + α. The slope of the trend line (β coefficient) was then used to quantify CoP signal error as a function of both sway amplitude and direction.

The WBB's performance was then quantified by comparing the CoP measures (Table 1) derived from both the *CoP_WBB_* and *CoP_AFP_′* signals (*measure_WBB_vs. measure_AFP_*). The CoP measure error was defined as the percent difference between AFP- and WBB-based CoP measures. This error was calculated for each CoP measure, treating the measures derived from the *CoP_AFP_′* signals as the ground truth:

(9) $$\text{CoP measure error}=\frac{100*({\text{measure}}_{\text{AFP}}-{\text{measure}}_{\text{WBB}})}{{\text{measure}}_{\text{AFP}}}$$

Bland-Altman plots were used to visually represent the WBB's CoP measure error.

#### 2.4.4. Linear Calibration of the *CoP_WBB_* Signals to Reduce the CoP Measurement Error

After characterizing the CoP signal error (detailed in Section 2.4.3), simple linear regression was implemented to linearly correct the *CoP_WBB_* signals and reduce measurement error. Simple linear regression was used to fit a straight trend line to the *CoP_WBB_* signals plotted against the *CoP_AFP_′* signals (*CoP_WBB_* = *m* × *CoP_AFP_′* + *b*). The linear regression coefficients (*m_AP_, b_AP_, m_ML_, b_ML_*) in Table 2 represent the statistical means averaged across all sway amplitudes, in both sway directions, for each of the 12 WBBs. These WBB-specific coefficients were then used to linearly calibrate all one-dimensional *CoP_WBB_* signals acquired from each WBB (*CoP_WBB_^calib^* = 1/*m* × (*CoP_WBB_* − *b*)). The linear regression coefficients stored in the last row of Table 2 represent the statistical means averaged across all 12 WBBs There was a statistical difference across directions: the slope of the trend lines (*m* coefficients) were significantly less in the ML direction (*p* < 0.001). However, there was no statistical difference between *m* coefficients across the 12 WBBs (*p* = 1 in both directions).

Because all CoP signals were zero-meaned, the trend line y-intercepts (*b_AP_* and *b_ML_*) should equal zero. The y-intercepts reported in Table 2 are not significantly different from zero and therefore should not influence the calibration procedure we recommend to use for any WBB. Only the mean trend line slopes (*m_AP_* and *m_ML_* in the last row of Table 2) should be used to linearly calibrate *CoP_WBB_* signals acquired from any WBB (*CoP_WBB_^calib^* = 1/*m* × (*CoP_WBB_*)).

#### 2.4.5. Statistical Analysis

First, the CoP signals were analyzed. For Aim I (validation), Pearson's linear correlation coefficients were calculated to assess CoP signal agreement (*CoP_WBB_vs*. *CoP_AFP_′*). Then, root-mean-squared errors (RMSE) (in mm) were calculated to quantify the difference between the *CoP_WBB_* and *CoP_AFP_′* signals. A t-test was performed to confirm that the RMSEs were significantly different from zero before calibration. To investigate the effect of direction on CoP signal error, one-way, fixed effect (sway direction) ANOVAs were performed on both the RMSEs and β coefficients (defined in Section 2.4.3). Then, to investigate the effect of calibration on signal error, one-way, fixed effect (calibration) ANOVAs were performed on the RMSEs in both sway directions. We then performed a one-way, fixed effect (sway direction) ANOVA on the RMSEs after calibration to investigate the effect of direction on signal error after linear calibration of the *CoP_WBB_* signals. For Aim II (inter-device variability), one-way, fixed effect (WBB) ANOVAs were performed on both the RMSEs and the β coefficients to assess the effect of the 12 WBBs on CoP signal error in both directions, before calibration. To assess inter-device variability after calibration, one-way, fixed effect (WBB) ANOVAs were performed on the RMSEs in both directions.

As a secondary analysis, we investigated the effect of sway amplitude and frequency on CoP signal error. Two-way, repeated measures, fixed effects (displacement angle, load height) ANOVAs were performed on the RMSEs to assess both the main and interaction effects of displacement angle and load height on signal error in both directions, both before and after calibration. A Bonferroni correction was applied to account for multiple comparisons (3 displacement angles × 3 load heights = 9 comparisons).

Second, the CoP measures were analyzed. For Aim I (validation), one-way, fixed effect (device) ANOVAs were first performed on all one-dimensional CoP measures (defined in Table 1) to assess the difference between AFP- and WBB-based CoP measures in both directions. To investigate the effect of direction on CoP measure error, one-way, fixed effect (sway direction) ANOVAs were performed on all CoP measure errors before calibration. Then, to investigate the effect of calibration on measure error, one-way, fixed effect (calibration) ANOVAs were performed on the CoP measure errors in both sway directions. We then performed one-way, fixed effect (sway direction) ANOVAs on all CoP measure errors after calibration to investigate the effect of direction after linear calibration of the *CoP_WBB_* signals. For Aim II (inter-device variability), a one-way fixed effect (WBB) ANOVA was performed for each measure to assess the effect of the 12 WBBs on CoP measure error in both directions, both before and after calibration.

## 3. Results and Discussion

### 3.1. CoP Signal Error

#### 3.1.1. CoP Signal Error before Linear Calibration of the *CoP_WBB_* Signals

**Figure 5.**
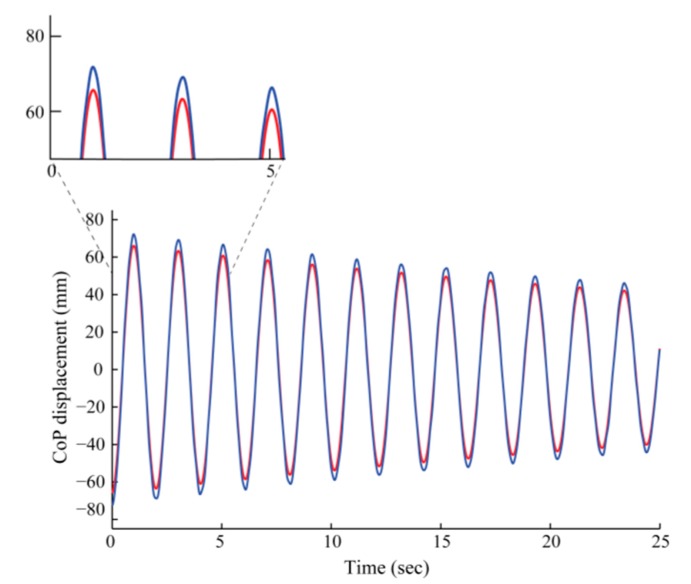
The *CoP_WBB_* (in blue) and *CoP_AFP_′* (in red) signals for the condition invoking the lowest frequency response and highest sway amplitude. The zoomed-in templates illustrate the WBB's CoP signal error: the difference (in mm) in CoP displacement (*CoP_WBB_* − *CoP_AFP_′*).

The *CoP_WBB_* signals were significantly correlated with the *CoP_AFP_* signals across all sway amplitudes and frequencies and in both sway directions for all 12 WBBs (*r* > 0.99) (Figure 5).

**Figure 6.**
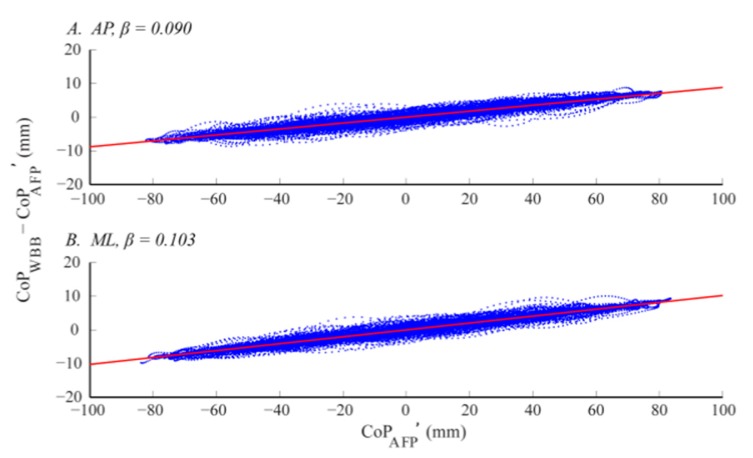
An individual WBB's (WBB_4) CoP signal error (*CoP_WBB_* − *CoP_AFP_′*) is plotted for all sway amplitudes and in both the AP (**A**) and ML (**B**) directions.

The CoP signal error was a function of CoP magnitude. As the sway amplitude increased the CoP signal error increased, indicated by positive slopes (*β_AP_, β_ML_*) of the linear trend lines (in red) in Figure 6. In other words, the WBB's accuracy appears to decrease as horizontal and shear sway components increase. As shown below in Figure 6, agreement between CoP signals was not only a function of sway amplitude but also a function of sway direction. The CoP signal error was larger in the ML direction, indicated by a steeper slope (*β_ML_*) in Figure 6B.

The *β* coefficients in Table 3 characterize the direction-specific slope of the linear trends for each WBB. The linear regression coefficients (*β_AP_, α_AP_, β_ML_, α_ML_*) were derived from the CoP signal error across all sway amplitudes, in both directions, for each of the 12 WBBs. There was a significant difference in signal error across directions: the β coefficients are significantly greater in the ML direction (*F_1,22_* = 24.30, *p* < 0.001). However, there was no statistical difference between β coefficients across the 12 WBBs, indicating low inter-device variability (*p* = 1 in both directions).

These findings were statistically supported by our analysis of RMSEs. As discussed in Section 2.4.5, RMSEs quantify residuals and represent the difference between the *CoP_WBB_* and *CoP_AFP_′* signals. The means and standard deviations of the RMSEs were 3.5 ± 0.9 mm and 4.0 ± 1.1 mm for the AP and ML directions, respectively. The RMSEs were significantly greater than zero (*p* < 0.001 in both directions), and the ML RMSEs were significantly greater than the AP RMSEs (*F_1,214_* = 15.19, *p* < 0.001). There was no statistically significant difference in RMSEs across the 12 WBBs, indicating low inter-device variability (AP: *F_11,96_* = 0.53, *p* = 0.881; ML: *F_11,96_* = 0.28, *p* < 0.988).

Additionally, there was a significant effect of displacement angle (*θ_i_* = 2°, 4°, and 6°) on RMSE, with a significant increase in RMSE as displacement angle increased (AP: *F_2,2,4_* = 234.46, *p* < 0.001; ML: *F_2,2,4_* = 232.79, *p* < 0.001). There was a significant effect of load height (*h* = 900, 1000 and 1100 mm) on RMSE in the AP direction (*F_2,2,4_* = 5.86, *p* < 0.004), with a significant decrease in RMSE as load height increased. There was not a significant effect of load height in the ML direction (*F_2,2,4_* = 0.05, *p* = 0.950) and there was no interaction between the two factors (displacement angle and load height). We hypothesize that there was no effect of load height on RMSE in the ML direction due to the larger magnitude and wider distribution of RMSE in the ML direction (see Figure 8, before calibration).

#### 3.1.2. CoP Signal Error after Linear Calibration of the *CoP_WBB_* Signals

**Figure 7.**
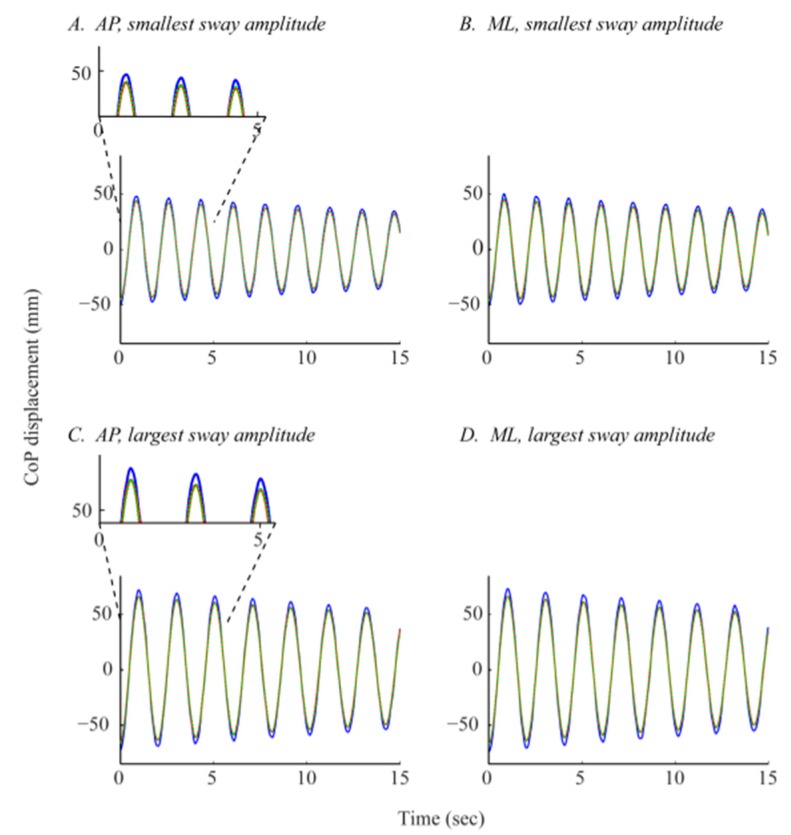
Effect of calibration on *CoP_WBB_* signals. All four plots contain three signals: The *CoP_WBB_* signal before calibration (in blue, solid line), the *CoP_WBB_* signal after calibration (*CoP_WBB_^calib^*) (in green, dashed line), and the “gold standard” *CoP_AFP_′* signal (in red, solid line).

The difference between the *CoP_WBB_* and *CoP_AFP_′* signals, quantified by the RMSEs, was significantly reduced by the linear calibration of the *CoP_WBB_* signals. Figure 7 shows the effect of calibration on the *CoP_WBB_* signals.

There was a significant reduction in RMSEs with calibration (AP: *F_1,214_* = 856.52, *p* < 0.001; ML: *F_1,214_* = 794.05, *p* < 0.001). After calibration, the RMSEs were no longer significantly greater in the ML direction (*F_1,214_* = 0.37, *p* = 0.5451). Similar to the results before calibration, there was no difference in RMSEs across the 12 WBBs (AP: *F_11,96_* = 0.11, *p* = 0.999; ML: *F_11,96_* = 0.24, *p* < 0.993). Linear calibration of the *CoP_WBB_* signals reduced the inter-device variability, which in turn strengthened the WBB's inter-device reliability.

**Figure 8.**
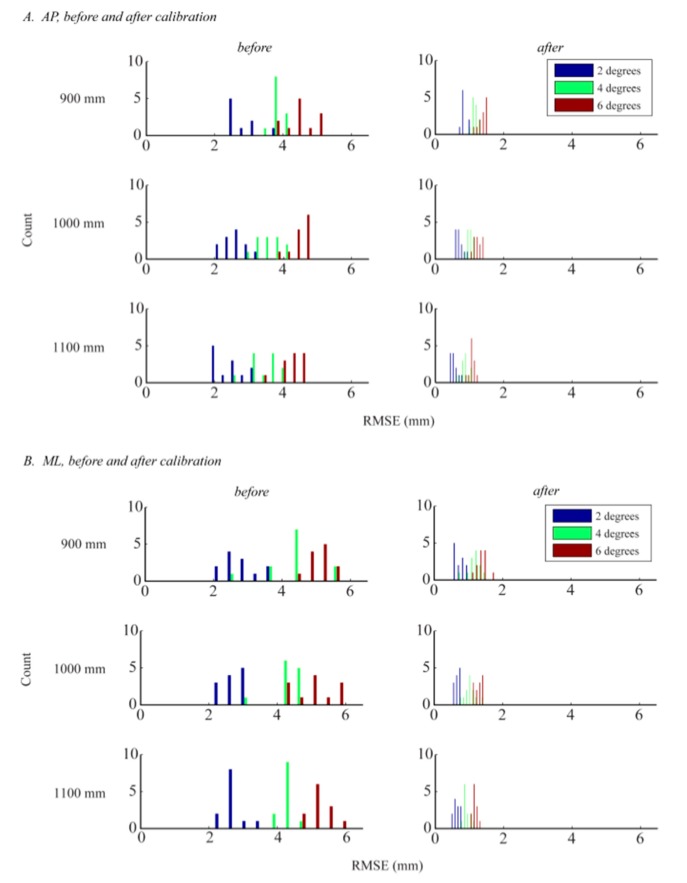
Effect of calibration on *CoP_WBB_* signal error measured by RMSEs. This figure shows the distribution of the RMSEs across all sway amplitudes and WBBs, in both the AP (**A**) and ML (**B**) directions, both before and after linear calibration of the *CoP_WBB_* signals. The three oscillation frequencies (*ω*) corresponding to the three load heights (*h* = 900, 1000 and 1100 mm) are 0.6, 0.5, and 0.4 Hz, respectively.

The significant effect of displacement angle remained after calibration. Like before, the RMSE values increased as displacement angle increased (AP: *F_2,2,4_* = 204.71, *p* < 0.001; ML: *F_2,2,4_* = 170.82, *p* < 0.001). As discussed in Section 3.1.1, there was a significant effect of load height in the AP but not in the ML direction before calibration. Because the CoP signal error was significantly greater in the ML direction before calibration, and because our linear calibration procedure corrects the CoP measurement and reduces error, there was an effect of load height in the ML direction after calibration. After calibration, the RMSEs significantly decreased as load height increased in both sway directions (AP: *F_2,2,4_* = 55.27, *p* < 0.001; ML: *F_2,2,4_* = 20.75, *p* < 0.001). These results are consistent with what we expected to see since larger displacement angles and shorter load heights produce larger CoP amplitudes (Section 2.4.1) and, as we saw in our primary analysis, RMSEs increase as sway amplitude increases. Like before, there was no interaction between the two factors (displacement angle and load height). The significant effect of calibration on CoP signal error is shown in Figure 8.

### 3.2. CoP Measure Error

#### 3.2.1. CoP Measure Error before Linear Calibration of the *CoP_WBB_* Signals

Before calibration, there was a significant difference between the AFP- and WBB-based time-domain measures, indicated by large *F* statistics and small *p* values in Table 4A. However, there was no difference between the AFP- and WBB-based frequency-domain measure, peak frequency (*PFREQ*), in both directions (Table 4A).

**Figure 9.**
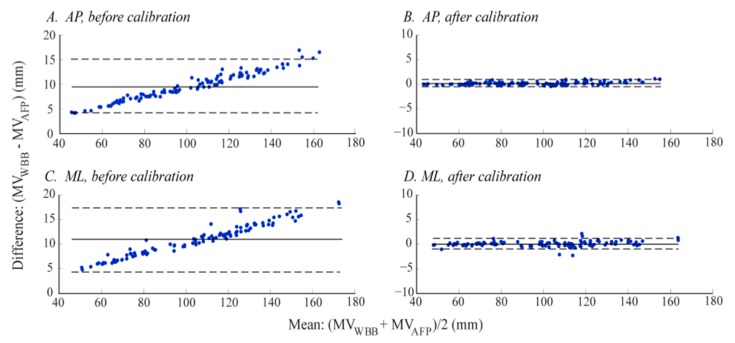
Bland-Altman plots of a time-domain measure, mean velocity (*MV*), before and after linear calibration of the *CoP_WBB_* signals. Comparison of *MV* derived from both the *CoP_WBB_* and *CoP_AFP_′* signals for every trial (12 WBBs, three load heights, three displacement angles per load height, per sway direction = 108 trials). The solid line represents the mean difference between measurements (*MV_WBB_vs. MV_AFP_*) and the dotted lines represent the 95% limits of agreement (±1.96 times the standard deviation of the mean difference).

The CoP measure error (defined in Section 2.4.3), averaged across all sway amplitudes and WBBs, was about −10% and −11% for all AP and ML time-domain measures, respectively (Table 5A). There was a significant difference in error between directions (AP *vs.* ML) for all CoP time-domain measures (*p* values in Figure 5A). Since the CoP measures were derived from the CoP signals, the error in *CoP_WBB_* time-domain measures was a direct function of the CoP signal error (discussed in Section 3.1.1), as seen in our Bland-Altman analysis. For example, the Bland-Altman plot for the CoP time-domain measure, mean velocity (*MV*), before calibration (Figure 9A,C) follows the same trend expressed by the CoP signal error (Figure 5). There was no CoP measure error for the frequency-domain measure, *PFREQ* (Table 5, A), meaning the WBB accurately measured the frequency content of the CoP signals.

There was a significant effect of WBB on CoP measure error *p* < 0.001 for all CoP time-domain measure errors, with error from one WBB (WBB_12) being significantly greater than the error from the remaining 11 WBBs in the AP direction and error from a different WBB (WBB_1) being significantly greater than the error from the remaining 11 WBBs in the ML direction). Evidently, there is significant inter-device variability on CoP measure error (based on the performance of two out of 12 different WBBs) before calibration.

#### 3.2.2. CoP Measure Error after Linear Calibration of the *CoP_WBB_* Signals

After calibration, there was no statistical difference between the AFP- and WBB-based time-domain measures, indicated by extremely small *F* statistics and large *p* values in Table 4B. Like before calibration, there was no difference between the AFP- and WBB-based *PFREQ* after calibration (Table 4B). Since our linear calibration simply corrects the CoP measurement (in mm) and reduces error, it has no effect on the frequency content of the CoP signal and in turn on the frequency-domain measure *PFREQ*.

The significant reduction of CoP time-domain measure errors (from Table 5A–B) shows the significant effect of calibration on *CoP_WBB_* measure accuracy in the time-domain (*p* < 0.001 for all CoP time-domain measure errors, in both directions). This effect is clearly illustrated in Figure 9: Before calibration there was a strong correlation between measure error and amplitude, with an increase in error as measure amplitude increases, but this effect was absent after calibration. After calibration, just one CoP time-domain measure remained sensitive to direction: The magnitude of error for *MV* was significantly greater in the AP direction (*p* values in Figure 5B). As expected, the CoP measure error for *PFREQ* did not change with calibration.

Dissimilar to the results before calibration, there was no effect of WBB on CoP measure error after calibration (0.930 < *p* ≤ 1.000 for all CoP time-domain measure errors, in both directions). These findings suggest that our proposed calibration procedure is effective when comparing CoP measures acquired from different WBBs.

## 4. Conclusions

The WBB is an affordable, portable, and easily accessible device that may be used to measure ground reaction forces and CoP displacement in situations where lower accuracy and precision is acceptable. The WBB should not be used as a replacement for the “gold standard” laboratory grade force-plate when measuring CoP under both static and dynamic conditions, as it is a uni-axial device and lacks the accuracy recommended for posturography [27]. However, when calibrated with the “gold standard,” as done in this study using an AMTI force plate (AFP), the WBB may be used to estimate time-domain CoP measures with improved accuracy. Linear calibration of the *CoP_WBB_* signal (detailed in Section 2.4.4) is recommended to reduce CoP measurement error and provide more reliable estimates of time-domain CoP measures.

The WBB's time jitter poses a significant limitation, in general, for the use of the WBB as a CoP measurement device. Because the WBB samples at an inconsistent rate, we employed a data averaging method to create time series with samples at equal time intervals (*t_DA_*). In this study, we averaged across samples every 93.75 ms (*t_DA_* = 0.09375 s; *f_DA_* = ∼10.7 Hz). Because we found the WBB's mean sampling rate *f_s_* to be ∼50 Hz, we could potentially increase CoP measurement accuracy and reduce error by decreasing the time interval *t_DA_*, in which we averaged across samples. However, occasionally the WBB only acquired 1 or 2 samples worth of data during the specified *t_DA_* (even though it usually acquired 3–6 samples, as discussed in Section 2.3). If our custom-written software was set to average across samples at a faster rate, *f_DA_*, our dataset could contain missing data. Because the WBB has been reported to sample at different mean rates (*f_s_* = 35 [16], 40 [15], 64 [21], and even 100 Hz [20]), we conclude that the WBB's mean sampling rate depends on both the device and the operating system of the device used to connect to the WBB. The WBB's time jitter combined with an inappropriately fast *f_DA_* (dependent upon the connected device) could explain the “occasional glitches in the data” reported in previous publications [16,21]. Lastly, an accurate frequency analysis is unobtainable due to the significant time jitter (>0.01 s) in the WBB data since most commonly used frequency analysis methods assume equally spaced samples in time [21]. In this study, we derived only one frequency-domain CoP measure—peak frequency, *PFREQ*—since, in theory, our controlled input signals only contained one frequency (the inverted pendulum's oscillation frequency (*ω*)). Although the WBB accurately measured *PFREQ* for our input signals, we do not recommend using the WBB to measure frequency content for clinical or research purposes since human postural sway is two- (or three-, if considering the vertical axis) dimensional and consists of a further distributed frequency spectrum. In sum, the device and operating system used to acquire data from the WBB, as well as the way in which the WBB's time jitter is treated, may affect the quality of CoP measures derived from WBB data.

The way in which we processed both the WBB- and AFP-based CoP signals poses another limitation of this study. As reported in Section 2.4.1, we used PCA to synchronize the *CoP_WBB_* and *CoP_AFP_′* signals in space (to account for the inherent yet small positioning errors) and we zero-meaned the signals when time-aligning offline. In doing so, we were unable to detect the WBB's initial (horizontal) offset error previously documented by Bartlett *et al.* [20]. Despite this limitation, our research findings remain significant with regards to purpose and implementation. Although we do not quantify the WBB's initial (horizontal) offset error, we do quantify the WBB's (horizontal) CoP measurement error once the *CoP_WBB_* and *CoP_AFP_′* signals are synchronized in both space and time. Our future research aims motivated the signal processing methods carried out in this study. In future work, we plan to derive summary postural sway measures (such as the CoP time-domain measures reported in Table 1) from human postural sway measured by a single WBB on a frequent, longitudinal basis. We are more concerned with the relative accuracy and reliability of WBB-based CoP measures and less concerned with the WBB's absolute precision as a CoP measurement device. Furthermore, assuming there is no drift in the WBB's (horizontal) offset error across time (a potential issue which has yet to be explored), it would not influence summary measures like mean sway amplitude, path length, and velocity.

Additional limitations of this study pertain to our experimental setup and procedures. The weight distribution of the inverted pendulum mechanical system does not closely resemble that of a human body, in which two thirds of the body's weight is concentrated at or around the height of the body's CoM [29]. Most of the mechanical system's weight was concentrated at the base with only 28% of the weight loaded at the approximate height of a human body's CoM. So although we acquired promising results, we cannot make confident inferences regarding the WBB's competency in practice until we test a load with a weight distribution more representative of a human body. Furthermore, our simulated postural sway signals (with respect to both sway amplitude and frequency) differ from human sway. As previously discussed, we systematically tested multiple displacement angles and load heights to induce a variety of sway amplitudes and frequencies. Although the load heights were selected according to the approximate height of a human body's CoM [25,26], the selected displacement angles induced higher sway amplitudes than typically seen human postural sway. We were restricted to a relatively large range of displacement angles (*θ_i_* = 2°, 4° and 6°) due to the limited precision of the device used to measure the displacement angles. If we were able to reduce and restrict our range (e.g., to *θ_i_* = 0.5°, 1.0° and 1.5°), we would have induced sway amplitudes that were more similar to human postural sway. We expect a lower CoP measurement error when using the WBB to measure human postural sway since human sway tends to be lower in amplitude relative to our simulated postural sway amplitudes and since we determined error to be a function of sway amplitude in this study. Lastly, we only assessed one-dimensional sway and a limited range of sway frequencies (*ω* = 0.4, 0.5 and 0.6 Hz induced by *h* = 1100, 1000 and 900 mm, respectively). The range of frequencies tested is comparable to the mean frequencies found in both healthy young and old adults during quiet stance with both eyes opened and eyes closed [30], however a higher frequency range (1–10 Hz) [31] should be tested in order to translate our findings to more challenging sway conditions that elicit an increase in postural sway (e.g., Romberg, standing on foam, or tandem stance) and/or to human populations with known postural instabilities and abnormal postural sway.

So although we have identified the WBB's CoP measurement error under controlled dynamic conditions, future work is needed before the WBB can be used as the sole postural sway measurement device in the clinical research and rehabilitation research domains. In agreement with Pagnacco *et al.* [22], we do not recommend the use of the WBB as a clinical diagnostic tool. The WBB was designed and manufactured for entertainment purposes and lacks the accuracy, precision, and reliability required of medical devices. In future work, human postural sway must be measured under a variety of sway conditions and in many human populations (differentiated by both age and health status) simultaneously with both the WBB and force plate in order to characterize CoP measurement error under uncontrolled dynamic conditions. Additionally, the WBB's performance over time (e.g., days, weeks, months, *etc.*) has yet to be assessed. Before the WBB can be used to frequently and longitudinally monitor postural sway, its CoP measurement error must be quantified and analyzed across an extended period of time. Once the WBB's CoP measurement error is fully characterized and accounted for, the WBB could substitute for a laboratory-grade force plate in situations where lower accuracy and precision is acceptable, such as for frequent, longitudinal monitoring of postural sway for older adults in a small clinic or home environment.

## Figures and Tables

**Table 1.** Time- and frequency-domain CoP measures derived from one-dimensional CoP signals.

**Measure**	**Abbr.**	**Description**	**Units**
***Time-Domain Measures***			
Mean distance, or sway amplitude	*MD*	Average distance from the center of the CoP time series	*mm*
Root-mean-squared distance	*RMS*	The standard deviation (SD) of the zero-meaned CoP time series	*mm*
Sway range	*RANGE*	Peak-to-peak range, or maximum distance, of the CoP values	*mm*
Mean velocity	*MV*	Average velocity of the CoP time series	*mm·s*^−^*^1^*
***Frequency-Domain Measure***			
Peak frequency	*PFREQ*	Peak frequency of the power spectrum	*Hz*

**Table 2.** The linear regression coefficients (*m_AP_, b_AP_, m_ML_, b_ML_*) used to calibrate all one-dimensional *CoP_WBB_* signals for each of the 12 WBBs. Simple linear regression was used to fit a straight trend line to the *CoP_WBB_* signals plotted against the *CoP_AFP_′* signals (*CoP_WBB_^calib^* = 1/*m* × (*CoP_WBB_* − *b*)) for each WBB.

**WBB**	**AP**		**ML**	

	***m****_AP_*	***b****_AP_*	***m****_ML_*	***b****_ML_*
WBB_1	1.087	−0.002	1.111	0.001
WBB_2	1.086	0.020	1.097	0.010
WBB_3	1.086	0.006	1.098	−0.020
WBB_4	1.084	−0.011	1.094	−0.040
WBB_5	1.085	−0.001	1.095	0.014
WBB_6	1.088	−0.019	1.096	0.029
WBB_7	1.091	0.002	1.097	0.020
WBB_8	1.093	−0.015	1.102	−0.010
WBB_9	1.086	0.020	1.094	0.012
WBB_10	1.090	−0.008	1.101	−0.005
WBB_11	1.085	0.005	1.093	−0.017
WBB_12	1.099	0.025	1.097	−0.008
**mean ± std**	**1.088 ± 0.004**	**0.002 ± 0.014**	**1.098 ± 0.005**	**−0.001 ± 0.019**

**Table 3.** The linear regression coefficients (*β_AP_, α_AP_, β_ML_, α_ML_*) were derived from the CoP signal error across all sway amplitudes and frequencies, in both directions, for each of the 12 WBBs. Simple linear regression was used to fit a straight trend line to the CoP signal error plotted against the *CoP_AFP_′* signals: *CoP_WBB_* − *CoP_AFP_′* = *β* × *CoP_AFP_′* + *α*.

**WBB**	**AP**		**ML**	

	***β****_AP_*	***α****_AP_*	***β****_ML_*	***α****_ML_*
WBB_1	0.094	−0.002	0.125	0.001
WBB_2	0.094	0.021	0.107	0.011
WBB_3	0.093	0.007	0.108	−0.022
WBB_4	0.090	−0.012	0.103	−0.044
WBB_5	0.092	−0.001	0.104	0.015
WBB_6	0.096	−0.021	0.105	0.032
WBB_7	0.099	0.003	0.107	0.022
WBB_8	0.102	−0.017	0.112	−0.011
WBB_9	0.094	0.022	0.103	0.013
WBB_10	0.098	−0.009	0.111	−0.006
WBB_11	0.092	0.006	0.101	−0.019
WBB_12	0.109	0.028	0.107	−0.009
**mean ± std**	**0.096 ± 0.005**	**0.002 ± 0.016**	**0.108 ± 0.006**	**−0.001 ± 0.022**

**Table 4.** Means and standard deviations of both AFP- and WBB-based CoP measures, both before and after linear calibration of the *CoP_WBB_* signals. Results from the one-way, fixed effects (device) ANOVAs shows the difference between AFP- and WBB-based CoP measures before and after linear calibration.

**A.** ***Before*** **Linear Calibration of** ***CoP****_WBB_* **Signals:**									
**Measure**	**Units**	**AP**				**ML**			
		**AFP: mean ± std**	**WBB: mean ± std**	***F****_1,214_*	***p*** **value**	**AFP: mean ± std**	**WBB: mean ± std**	***F****_1,214_*	***p*** **value**
***Time-Domain Measures***									
*MD*	*mm*	31.0 ± 7.8	34.0 ± 8.5	7.23	0.008	32.2 ± 8.8	35.6 ± 9.7	7.64	0.006
*RMS*	*mm*	34.9 ± 8.8	38.2 ± 9.6	7.32	0.007	36.1 ± 9.8	40.0 ± 10.8	7.71	0.006
*RANGE*	*mm*	123.5 ± 30.4	135.6 ± 33.2	7.82	0.006	128.3 ± 34.4	142.2 ± 38.0	7.97	0.005
*MV*	*mm·s^−1^*	97.2 ± 27.1	106.8 ± 29.9	6.16	0.014	100.0 ± 28.4	110.9 ± 31.6	7.14	0.008
***Frequency-Domain Measure***									
*PFREQ*	*Hz*	0.5 ± 0.1	0.5 ± 0.1	0.00	1.000	0.5 ± 0.1	0.5 ± 0.1	0.00	1.000
**B.** ***After*** **Linear Calibration of** ***CoP****_WBB_* **Signals:**									
**Measure**	**Units**	**AP**				**ML**			
		**AFP: mean ± std**	**WBB: mean ± std**	***F****_1,214_*	***p* value**	**AFP: mean ± std**	**WBB: mean ± std**	***F****_1,214_*	***p*** **value**
***Time-Domain Measures***									
*MD*	*mm*	31.0 ± 7.8	31.0 ± 7.8	<0.001	0.993	32.2 ± 8.8	32.1 ± 8.8	<0.001	0.996
*RMS*	*mm*	34.9 ± 8.8	34.9 ± 8.7	<0.001	0.998	36.1 ± 9.8	36.1 ± 9.8	<0.001	0.998
*RANGE*	*mm*	123.5 ± 30.4	123.6 ± 30.3	<0.001	0.975	128.3 ± 34.4	128.3 ± 34.4	<0.001	0.994
*MV*	*mm·s^−1^*	97.2 ± 27.1	97.4 ± 27.3	<0.001	0.957	100.0 ± 28.4	100.0 ± 28.5	<0.001	0.983
***Frequency-Domain Measure***									
*PFREQ*	*Hz*	0.5 ± 0.1	0.5 ± 0.1	0.00	1.000	0.5 ± 0.1	0.5 ± 0.1	0.00	1.000

**Table 5.** CoP time-domain measure (%) errors both before and after linear calibration of *CoP_WBB_* signals. The *p* values quantify the direction-specific difference in error (AP *vs.* ML) both before (**A**) and after (**B**) calibration. The (%) errors for the one CoP frequency-domain measure *PFREQ* is not reported here because there is no difference in *PFREQ* measured by both the WBB and AFP (see Table 4).

**Measures**	**A. CoP Measure (%) Error** ***before*** **Linear Calibration of** ***CoP****_WBB_* **Signals:**					**B. CoP Measure (%) Error** ***after*** **Linear Calibration of** ***CoP****_WBB_* **Signals:**		
	**AP mean** ± **std**		**ML mean** ± **std**	***p*** **value**	**AP mean** ± **std**		**ML mean** ± **std**	*p* **value**
***Time-Domain Measures***								
*MD*	−9.70 ± 0.67	−10.85 ± 0.88		<0.001	−0.01 ± 0.40		0.01 ± 0.57	0.805
*RMS*	−9.71 ± 0.64	−10.86 ± 0.88		<0.001	−0.02 ± 0.37		0.00 ± 0.56	0.744
*RANGE*	−9.83 ± 0.76	−10.90 ± 0.91		<0.001	−0.13 ± 0.57		−0.03 ± 0.64	0.251
*MV*	−9.89 ± 0.60	−10.90 ± 0.87		<0.001	−0.18 ± 0.34		−0.04 ± 0.56	0.021
